# What Predicts Advance Care Planning Among Older Adults: Perceived Health or the Number of Chronic Conditions?

**DOI:** 10.1093/geroni/igaa057.1358

**Published:** 2020-12-16

**Authors:** YuHsuan (Olivia) Wang, Susan Enguidanos, Olivia Wang

**Affiliations:** University of Southern California, Los Angeles, California, United States

## Abstract

Advance care planning (ACP) is associated with improved quality of death and better end-of-life care. Studies have found that both health status and chronic illness influence rates of ACP. However, little is known about the relative association of each factor with engaging in ACP. This study aims to identify the extent to which the number of chronic conditions and self-rated health predict engaging in ACP. We used data from the Health and Retirement Study, a nationally-representative longitudinal survey of older adults. The sample consisted of 2016 core interview respondents. We conducted logistic regression models to examine the association between self-rated health and the number of self-reported chronic conditions with three dependent variables: (1) ACP engagement (n=687), (2) AD completion (n=1671), and (3) assignment of health care proxy (n=1668), while controlling for demographic characteristics,. Samples were weighted. Analysis revealed that reporting more chronic conditions was associated with higher odds of advance directive completion (OR:1.21, p<.001), ACP engagement (OR: 1.26, p<.05), and assigning medical proxies (OR: 1.32, p<.001). However, better self-reported health was associated with higher odds of having an AD (OR: 1.20, p<.05) and assigning medical proxies (OR: 1.27, p<.01). These findings suggest that greater number of chronic conditions increased the odds of having an AD, engaging in ACP and in assigning medical proxies; however, those reporting better health were more likely to have an AD and a medical proxy. Findings from this study point suggest that individuals with multi-morbidities may be more open to engaging in ACP discussions and activities.

